# Supplementary material for: Multi‐dataset identification of innovative feature genes and molecular mechanisms in keratoconus
Source: J Cell Mol Med. 2024 Sep 19;28(18):e70079. doi: 10.1111/jcmm.70079 (PMC11412914; doi:10.1111/jcmm.70079)
Supplement: Supplementary file 2 — Material S2. [file JCMM-28-e70079-s002.zip › 7.GSEA analysis/6.SNORA74B/gsea_report_for_l_1678852949716.html]

Report for l 1678852949716 [GSEA]

| GS  follow link to MSigDB | GS DETAILS | SIZE | ES | NES | NOM p-val | FDR q-val | FWER p-val | RANK AT MAX | LEADING EDGE || 1 | KEGG\_BASAL\_CELL\_CARCINOMA | Details ... | 55 | -0.59 | -1.74 | 0.000 | 0.593 | 0.286 | 8112 | tags=44%, list=23%, signal=57% |
| 2 | KEGG\_FC\_GAMMA\_R\_MEDIATED\_PHAGOCYTOSIS | Details ... | 92 | -0.42 | -1.71 | 0.004 | 0.410 | 0.361 | 5459 | tags=21%, list=16%, signal=24% |
| 3 | KEGG\_INOSITOL\_PHOSPHATE\_METABOLISM | Details ... | 54 | -0.44 | -1.70 | 0.010 | 0.311 | 0.387 | 10322 | tags=39%, list=30%, signal=55% |
| 4 | KEGG\_PROSTATE\_CANCER | Details ... | 89 | -0.40 | -1.69 | 0.002 | 0.246 | 0.404 | 984 | tags=9%, list=3%, signal=9% |
| 5 | KEGG\_EPITHELIAL\_CELL\_SIGNALING\_IN\_HELICOBACTER\_PYLORI\_INFECTION | Details ... | 66 | -0.48 | -1.68 | 0.020 | 0.231 | 0.443 | 6589 | tags=26%, list=19%, signal=32% |
| 6 | KEGG\_CHEMOKINE\_SIGNALING\_PATHWAY | Details ... | 183 | -0.47 | -1.63 | 0.002 | 0.304 | 0.545 | 7046 | tags=31%, list=20%, signal=39% |
| 7 | KEGG\_ADHERENS\_JUNCTION | Details ... | 68 | -0.42 | -1.62 | 0.037 | 0.293 | 0.576 | 7593 | tags=24%, list=22%, signal=30% |
| 8 | KEGG\_MELANOGENESIS | Details ... | 101 | -0.43 | -1.62 | 0.018 | 0.261 | 0.579 | 4083 | tags=21%, list=12%, signal=23% |
| 9 | KEGG\_GLYCOSAMINOGLYCAN\_BIOSYNTHESIS\_CHONDROITIN\_SULFATE | Details ... | 22 | -0.60 | -1.61 | 0.022 | 0.262 | 0.596 | 7560 | tags=41%, list=22%, signal=52% |
| 10 | KEGG\_THYROID\_CANCER | Details ... | 29 | -0.44 | -1.58 | 0.028 | 0.307 | 0.651 | 7545 | tags=28%, list=22%, signal=35% |
| 11 | KEGG\_FC\_EPSILON\_RI\_SIGNALING\_PATHWAY | Details ... | 78 | -0.44 | -1.55 | 0.008 | 0.347 | 0.699 | 4251 | tags=21%, list=12%, signal=23% |
| 12 | KEGG\_TYROSINE\_METABOLISM | Details ... | 40 | -0.47 | -1.54 | 0.008 | 0.337 | 0.712 | 2233 | tags=23%, list=6%, signal=24% |
| 13 | KEGG\_ETHER\_LIPID\_METABOLISM | Details ... | 29 | -0.61 | -1.54 | 0.036 | 0.317 | 0.716 | 2270 | tags=21%, list=7%, signal=22% |
| 14 | KEGG\_PATHWAYS\_IN\_CANCER | Details ... | 322 | -0.36 | -1.52 | 0.019 | 0.331 | 0.737 | 9535 | tags=32%, list=27%, signal=43% |
| 15 | KEGG\_CELL\_CYCLE | Details ... | 124 | -0.42 | -1.52 | 0.098 | 0.323 | 0.742 | 10060 | tags=38%, list=29%, signal=53% |
| 16 | KEGG\_GLYCOLYSIS\_GLUCONEOGENESIS | Details ... | 61 | -0.42 | -1.51 | 0.029 | 0.317 | 0.755 | 11983 | tags=52%, list=35%, signal=80% |
| 17 | KEGG\_CELL\_ADHESION\_MOLECULES\_CAMS | Details ... | 130 | -0.47 | -1.51 | 0.017 | 0.313 | 0.768 | 7367 | tags=35%, list=21%, signal=44% |
| 18 | KEGG\_ARRHYTHMOGENIC\_RIGHT\_VENTRICULAR\_CARDIOMYOPATHY\_ARVC | Details ... | 74 | -0.49 | -1.50 | 0.029 | 0.312 | 0.776 | 7545 | tags=43%, list=22%, signal=55% |
| 19 | KEGG\_CHRONIC\_MYELOID\_LEUKEMIA | Details ... | 73 | -0.38 | -1.48 | 0.081 | 0.348 | 0.815 | 8196 | tags=30%, list=24%, signal=39% |
| 20 | KEGG\_TGF\_BETA\_SIGNALING\_PATHWAY | Details ... | 85 | -0.44 | -1.47 | 0.044 | 0.342 | 0.821 | 5878 | tags=29%, list=17%, signal=35% |
| 21 | KEGG\_PURINE\_METABOLISM | Details ... | 151 | -0.33 | -1.47 | 0.055 | 0.328 | 0.823 | 6511 | tags=21%, list=19%, signal=25% |
| 22 | KEGG\_ACUTE\_MYELOID\_LEUKEMIA | Details ... | 57 | -0.36 | -1.47 | 0.069 | 0.315 | 0.824 | 8368 | tags=32%, list=24%, signal=42% |
| 23 | KEGG\_MAPK\_SIGNALING\_PATHWAY | Details ... | 265 | -0.36 | -1.47 | 0.027 | 0.303 | 0.824 | 9219 | tags=33%, list=27%, signal=45% |
| 24 | KEGG\_TIGHT\_JUNCTION | Details ... | 127 | -0.40 | -1.47 | 0.032 | 0.297 | 0.827 | 7007 | tags=26%, list=20%, signal=32% |
| 25 | KEGG\_HUNTINGTONS\_DISEASE | Details ... | 158 | -0.26 | -1.45 | 0.082 | 0.324 | 0.851 | 15216 | tags=44%, list=44%, signal=78% |
| 26 | KEGG\_ENDOCYTOSIS | Details ... | 179 | -0.27 | -1.44 | 0.063 | 0.326 | 0.861 | 4503 | tags=10%, list=13%, signal=11% |
| 27 | KEGG\_PRION\_DISEASES | Details ... | 35 | -0.50 | -1.44 | 0.049 | 0.321 | 0.866 | 4428 | tags=29%, list=13%, signal=33% |
| 28 | KEGG\_SYSTEMIC\_LUPUS\_ERYTHEMATOSUS | Details ... | 56 | -0.58 | -1.44 | 0.078 | 0.312 | 0.867 | 4844 | tags=39%, list=14%, signal=46% |
| 29 | KEGG\_INTESTINAL\_IMMUNE\_NETWORK\_FOR\_IGA\_PRODUCTION | Details ... | 48 | -0.54 | -1.44 | 0.044 | 0.302 | 0.867 | 6958 | tags=46%, list=20%, signal=57% |
| 30 | KEGG\_NEUROTROPHIN\_SIGNALING\_PATHWAY | Details ... | 125 | -0.29 | -1.43 | 0.091 | 0.313 | 0.876 | 8211 | tags=22%, list=24%, signal=29% |
| 31 | KEGG\_NITROGEN\_METABOLISM | Details ... | 23 | -0.55 | -1.41 | 0.066 | 0.328 | 0.884 | 8080 | tags=48%, list=23%, signal=62% |
| 32 | KEGG\_PYRUVATE\_METABOLISM | Details ... | 40 | -0.36 | -1.41 | 0.118 | 0.322 | 0.887 | 10454 | tags=33%, list=30%, signal=46% |
| 33 | KEGG\_TOLL\_LIKE\_RECEPTOR\_SIGNALING\_PATHWAY | Details ... | 96 | -0.41 | -1.40 | 0.093 | 0.330 | 0.895 | 6257 | tags=29%, list=18%, signal=35% |
| 34 | KEGG\_ASTHMA | Details ... | 29 | -0.63 | -1.39 | 0.091 | 0.341 | 0.908 | 4452 | tags=41%, list=13%, signal=47% |
| 35 | KEGG\_GLUTATHIONE\_METABOLISM | Details ... | 49 | -0.41 | -1.39 | 0.143 | 0.339 | 0.910 | 12751 | tags=49%, list=37%, signal=77% |
| 36 | KEGG\_DNA\_REPLICATION | Details ... | 36 | -0.41 | -1.38 | 0.116 | 0.339 | 0.912 | 12464 | tags=72%, list=36%, signal=113% |
| 37 | KEGG\_LYSINE\_DEGRADATION | Details ... | 39 | -0.42 | -1.38 | 0.178 | 0.344 | 0.918 | 10127 | tags=38%, list=29%, signal=54% |
| 38 | KEGG\_CYTOKINE\_CYTOKINE\_RECEPTOR\_INTERACTION | Details ... | 256 | -0.46 | -1.37 | 0.041 | 0.346 | 0.918 | 7680 | tags=44%, list=22%, signal=56% |
| 39 | KEGG\_O\_GLYCAN\_BIOSYNTHESIS | Details ... | 29 | -0.48 | -1.37 | 0.131 | 0.344 | 0.920 | 3139 | tags=21%, list=9%, signal=23% |
| 40 | KEGG\_PANTOTHENATE\_AND\_COA\_BIOSYNTHESIS | Details ... | 16 | -0.52 | -1.37 | 0.139 | 0.337 | 0.920 | 7564 | tags=31%, list=22%, signal=40% |
| 41 | KEGG\_STEROID\_HORMONE\_BIOSYNTHESIS | Details ... | 46 | -0.44 | -1.36 | 0.043 | 0.339 | 0.925 | 4274 | tags=33%, list=12%, signal=37% |
| 42 | KEGG\_ADIPOCYTOKINE\_SIGNALING\_PATHWAY | Details ... | 66 | -0.39 | -1.36 | 0.135 | 0.332 | 0.925 | 10501 | tags=42%, list=30%, signal=61% |
| 43 | KEGG\_REGULATION\_OF\_ACTIN\_CYTOSKELETON | Details ... | 210 | -0.32 | -1.36 | 0.052 | 0.325 | 0.925 | 8006 | tags=24%, list=23%, signal=31% |
| 44 | KEGG\_GLYCOSPHINGOLIPID\_BIOSYNTHESIS\_LACTO\_AND\_NEOLACTO\_SERIES | Details ... | 26 | -0.47 | -1.35 | 0.099 | 0.330 | 0.930 | 516 | tags=12%, list=1%, signal=12% |
| 45 | KEGG\_WNT\_SIGNALING\_PATHWAY | Details ... | 149 | -0.32 | -1.35 | 0.091 | 0.325 | 0.930 | 3129 | tags=13%, list=9%, signal=15% |
| 46 | KEGG\_AXON\_GUIDANCE | Details ... | 128 | -0.35 | -1.35 | 0.083 | 0.318 | 0.930 | 6850 | tags=25%, list=20%, signal=31% |
| 47 | KEGG\_GLYCOSAMINOGLYCAN\_BIOSYNTHESIS\_KERATAN\_SULFATE | Details ... | 15 | -0.48 | -1.35 | 0.143 | 0.315 | 0.931 | 7639 | tags=47%, list=22%, signal=60% |
| 48 | KEGG\_PHOSPHATIDYLINOSITOL\_SIGNALING\_SYSTEM | Details ... | 76 | -0.32 | -1.35 | 0.129 | 0.315 | 0.931 | 1876 | tags=8%, list=5%, signal=8% |
| 49 | KEGG\_FOCAL\_ADHESION | Details ... | 196 | -0.39 | -1.34 | 0.136 | 0.311 | 0.931 | 8127 | tags=30%, list=23%, signal=38% |
| 50 | KEGG\_BUTANOATE\_METABOLISM | Details ... | 34 | -0.46 | -1.34 | 0.145 | 0.312 | 0.934 | 4108 | tags=18%, list=12%, signal=20% |
| 51 | KEGG\_LEISHMANIA\_INFECTION |  | 71 | -0.47 | -1.34 | 0.154 | 0.310 | 0.936 | 7344 | tags=38%, list=21%, signal=48% |
| 52 | KEGG\_MELANOMA |  | 71 | -0.39 | -1.33 | 0.107 | 0.312 | 0.944 | 381 | tags=8%, list=1%, signal=9% |
| 53 | KEGG\_GRAFT\_VERSUS\_HOST\_DISEASE |  | 39 | -0.53 | -1.33 | 0.159 | 0.317 | 0.947 | 4428 | tags=28%, list=13%, signal=32% |
| 54 | KEGG\_TYPE\_II\_DIABETES\_MELLITUS |  | 47 | -0.44 | -1.32 | 0.122 | 0.322 | 0.949 | 3174 | tags=19%, list=9%, signal=21% |
| 55 | KEGG\_ALZHEIMERS\_DISEASE |  | 144 | -0.25 | -1.31 | 0.106 | 0.325 | 0.952 | 3067 | tags=6%, list=9%, signal=7% |
| 56 | KEGG\_VIRAL\_MYOCARDITIS |  | 70 | -0.42 | -1.31 | 0.150 | 0.322 | 0.952 | 7007 | tags=29%, list=20%, signal=36% |
| 57 | KEGG\_RENAL\_CELL\_CARCINOMA |  | 66 | -0.42 | -1.31 | 0.139 | 0.317 | 0.952 | 9895 | tags=42%, list=28%, signal=59% |
| 58 | KEGG\_DORSO\_VENTRAL\_AXIS\_FORMATION |  | 24 | -0.42 | -1.31 | 0.186 | 0.312 | 0.953 | 4820 | tags=21%, list=14%, signal=24% |
| 59 | KEGG\_FRUCTOSE\_AND\_MANNOSE\_METABOLISM |  | 32 | -0.47 | -1.31 | 0.200 | 0.314 | 0.953 | 11881 | tags=53%, list=34%, signal=81% |
| 60 | KEGG\_METABOLISM\_OF\_XENOBIOTICS\_BY\_CYTOCHROME\_P450 |  | 60 | -0.41 | -1.31 | 0.096 | 0.310 | 0.953 | 4361 | tags=27%, list=13%, signal=30% |
| 61 | KEGG\_COLORECTAL\_CANCER |  | 62 | -0.34 | -1.30 | 0.168 | 0.308 | 0.953 | 7593 | tags=21%, list=22%, signal=27% |
| 62 | KEGG\_BLADDER\_CANCER |  | 41 | -0.46 | -1.29 | 0.232 | 0.317 | 0.960 | 9535 | tags=32%, list=27%, signal=44% |
| 63 | KEGG\_SPHINGOLIPID\_METABOLISM |  | 35 | -0.38 | -1.29 | 0.153 | 0.320 | 0.962 | 6966 | tags=29%, list=20%, signal=36% |
| 64 | KEGG\_INSULIN\_SIGNALING\_PATHWAY |  | 136 | -0.30 | -1.29 | 0.163 | 0.315 | 0.962 | 6814 | tags=18%, list=20%, signal=22% |
| 65 | KEGG\_HEDGEHOG\_SIGNALING\_PATHWAY |  | 55 | -0.41 | -1.29 | 0.102 | 0.316 | 0.962 | 3129 | tags=22%, list=9%, signal=24% |
| 66 | KEGG\_BASAL\_TRANSCRIPTION\_FACTORS |  | 33 | -0.30 | -1.28 | 0.205 | 0.316 | 0.962 | 13724 | tags=58%, list=40%, signal=95% |
| 67 | KEGG\_VASCULAR\_SMOOTH\_MUSCLE\_CONTRACTION |  | 111 | -0.36 | -1.27 | 0.128 | 0.326 | 0.965 | 8052 | tags=29%, list=23%, signal=37% |
| 68 | KEGG\_P53\_SIGNALING\_PATHWAY |  | 66 | -0.33 | -1.25 | 0.224 | 0.367 | 0.977 | 8045 | tags=27%, list=23%, signal=35% |
| 69 | KEGG\_ALDOSTERONE\_REGULATED\_SODIUM\_REABSORPTION |  | 42 | -0.37 | -1.24 | 0.151 | 0.365 | 0.979 | 5277 | tags=24%, list=15%, signal=28% |
| 70 | KEGG\_PYRIMIDINE\_METABOLISM |  | 95 | -0.28 | -1.24 | 0.247 | 0.374 | 0.982 | 13269 | tags=48%, list=38%, signal=78% |
| 71 | KEGG\_NOD\_LIKE\_RECEPTOR\_SIGNALING\_PATHWAY |  | 61 | -0.39 | -1.23 | 0.274 | 0.372 | 0.982 | 6884 | tags=26%, list=20%, signal=33% |
| 72 | KEGG\_PANCREATIC\_CANCER |  | 69 | -0.29 | -1.23 | 0.207 | 0.376 | 0.985 | 8497 | tags=25%, list=24%, signal=33% |
| 73 | KEGG\_RIG\_I\_LIKE\_RECEPTOR\_SIGNALING\_PATHWAY |  | 64 | -0.33 | -1.22 | 0.184 | 0.381 | 0.986 | 6754 | tags=22%, list=19%, signal=27% |
| 74 | KEGG\_PARKINSONS\_DISEASE |  | 98 | -0.29 | -1.21 | 0.279 | 0.390 | 0.990 | 1472 | tags=3%, list=4%, signal=3% |
| 75 | KEGG\_ALLOGRAFT\_REJECTION |  | 37 | -0.51 | -1.21 | 0.252 | 0.393 | 0.991 | 6813 | tags=38%, list=20%, signal=47% |
| 76 | KEGG\_LEUKOCYTE\_TRANSENDOTHELIAL\_MIGRATION |  | 114 | -0.32 | -1.20 | 0.137 | 0.398 | 0.991 | 7187 | tags=24%, list=21%, signal=30% |
| 77 | KEGG\_HYPERTROPHIC\_CARDIOMYOPATHY\_HCM |  | 83 | -0.36 | -1.20 | 0.197 | 0.397 | 0.991 | 7312 | tags=35%, list=21%, signal=44% |
| 78 | KEGG\_LONG\_TERM\_DEPRESSION |  | 67 | -0.36 | -1.19 | 0.197 | 0.403 | 0.992 | 8974 | tags=34%, list=26%, signal=46% |
| 79 | KEGG\_GALACTOSE\_METABOLISM |  | 25 | -0.39 | -1.19 | 0.277 | 0.403 | 0.993 | 11881 | tags=48%, list=34%, signal=73% |
| 80 | KEGG\_JAK\_STAT\_SIGNALING\_PATHWAY |  | 151 | -0.35 | -1.19 | 0.225 | 0.399 | 0.993 | 8003 | tags=37%, list=23%, signal=48% |
| 81 | KEGG\_AMINO\_SUGAR\_AND\_NUCLEOTIDE\_SUGAR\_METABOLISM |  | 42 | -0.34 | -1.19 | 0.304 | 0.398 | 0.993 | 10025 | tags=33%, list=29%, signal=47% |
| 82 | KEGG\_VIBRIO\_CHOLERAE\_INFECTION |  | 51 | -0.28 | -1.18 | 0.236 | 0.399 | 0.993 | 2679 | tags=10%, list=8%, signal=11% |
| 83 | KEGG\_CYTOSOLIC\_DNA\_SENSING\_PATHWAY |  | 47 | -0.35 | -1.18 | 0.237 | 0.397 | 0.994 | 6257 | tags=26%, list=18%, signal=31% |
| 84 | KEGG\_T\_CELL\_RECEPTOR\_SIGNALING\_PATHWAY |  | 107 | -0.32 | -1.17 | 0.227 | 0.412 | 0.995 | 6850 | tags=25%, list=20%, signal=31% |
| 85 | KEGG\_ONE\_CARBON\_POOL\_BY\_FOLATE |  | 17 | -0.42 | -1.17 | 0.300 | 0.409 | 0.995 | 11742 | tags=47%, list=34%, signal=71% |
| 86 | KEGG\_NATURAL\_KILLER\_CELL\_MEDIATED\_CYTOTOXICITY |  | 125 | -0.31 | -1.17 | 0.210 | 0.409 | 0.995 | 7536 | tags=30%, list=22%, signal=39% |
| 87 | KEGG\_LYSOSOME |  | 117 | -0.22 | -1.16 | 0.319 | 0.417 | 0.996 | 11819 | tags=29%, list=34%, signal=44% |
| 88 | KEGG\_ENDOMETRIAL\_CANCER |  | 52 | -0.30 | -1.16 | 0.297 | 0.414 | 0.997 | 7545 | tags=19%, list=22%, signal=25% |
| 89 | KEGG\_ALANINE\_ASPARTATE\_AND\_GLUTAMATE\_METABOLISM |  | 30 | -0.36 | -1.15 | 0.284 | 0.417 | 0.997 | 7540 | tags=37%, list=22%, signal=47% |
| 90 | KEGG\_APOPTOSIS |  | 87 | -0.29 | -1.15 | 0.279 | 0.423 | 0.998 | 6823 | tags=21%, list=20%, signal=26% |
| 91 | KEGG\_ARACHIDONIC\_ACID\_METABOLISM |  | 57 | -0.34 | -1.15 | 0.214 | 0.421 | 0.998 | 5345 | tags=23%, list=15%, signal=27% |
| 92 | KEGG\_GAP\_JUNCTION |  | 86 | -0.26 | -1.14 | 0.177 | 0.419 | 0.998 | 4812 | tags=14%, list=14%, signal=16% |
| 93 | KEGG\_PATHOGENIC\_ESCHERICHIA\_COLI\_INFECTION |  | 54 | -0.26 | -1.14 | 0.329 | 0.418 | 0.998 | 3825 | tags=9%, list=11%, signal=10% |
| 94 | KEGG\_PRIMARY\_BILE\_ACID\_BIOSYNTHESIS |  | 16 | -0.51 | -1.14 | 0.297 | 0.414 | 0.998 | 6720 | tags=50%, list=19%, signal=62% |
| 95 | KEGG\_B\_CELL\_RECEPTOR\_SIGNALING\_PATHWAY |  | 75 | -0.28 | -1.14 | 0.250 | 0.411 | 0.998 | 6850 | tags=24%, list=20%, signal=30% |
| 96 | KEGG\_PROXIMAL\_TUBULE\_BICARBONATE\_RECLAMATION |  | 23 | -0.38 | -1.14 | 0.247 | 0.411 | 0.998 | 9715 | tags=43%, list=28%, signal=60% |
| 97 | KEGG\_VASOPRESSIN\_REGULATED\_WATER\_REABSORPTION |  | 44 | -0.32 | -1.14 | 0.312 | 0.408 | 0.998 | 14733 | tags=61%, list=42%, signal=106% |
| 98 | KEGG\_GLYCEROPHOSPHOLIPID\_METABOLISM |  | 70 | -0.30 | -1.14 | 0.277 | 0.405 | 0.998 | 776 | tags=7%, list=2%, signal=7% |
| 99 | KEGG\_DRUG\_METABOLISM\_CYTOCHROME\_P450 |  | 62 | -0.34 | -1.13 | 0.295 | 0.406 | 0.998 | 4361 | tags=19%, list=13%, signal=22% |
| 100 | KEGG\_DILATED\_CARDIOMYOPATHY |  | 90 | -0.31 | -1.13 | 0.240 | 0.409 | 0.998 | 7312 | tags=32%, list=21%, signal=41% |
| 101 | KEGG\_HISTIDINE\_METABOLISM |  | 28 | -0.43 | -1.12 | 0.322 | 0.414 | 0.999 | 8067 | tags=36%, list=23%, signal=46% |
| 102 | KEGG\_TRYPTOPHAN\_METABOLISM |  | 39 | -0.34 | -1.12 | 0.256 | 0.413 | 0.999 | 3348 | tags=15%, list=10%, signal=17% |
| 103 | KEGG\_SMALL\_CELL\_LUNG\_CANCER |  | 84 | -0.29 | -1.12 | 0.320 | 0.409 | 0.999 | 8749 | tags=30%, list=25%, signal=40% |
| 104 | KEGG\_NICOTINATE\_AND\_NICOTINAMIDE\_METABOLISM |  | 24 | -0.41 | -1.11 | 0.381 | 0.420 | 0.999 | 5492 | tags=29%, list=16%, signal=35% |
| 105 | KEGG\_AMYOTROPHIC\_LATERAL\_SCLEROSIS\_ALS |  | 53 | -0.31 | -1.11 | 0.293 | 0.417 | 0.999 | 4026 | tags=15%, list=12%, signal=17% |
| 106 | KEGG\_COMPLEMENT\_AND\_COAGULATION\_CASCADES |  | 69 | -0.34 | -1.11 | 0.289 | 0.418 | 0.999 | 4646 | tags=26%, list=13%, signal=30% |
| 107 | KEGG\_NON\_SMALL\_CELL\_LUNG\_CANCER |  | 54 | -0.24 | -1.11 | 0.311 | 0.417 | 0.999 | 1107 | tags=6%, list=3%, signal=6% |
| 108 | KEGG\_CARDIAC\_MUSCLE\_CONTRACTION |  | 72 | -0.29 | -1.10 | 0.275 | 0.420 | 0.999 | 1885 | tags=11%, list=5%, signal=12% |
| 109 | KEGG\_TYPE\_I\_DIABETES\_MELLITUS |  | 43 | -0.41 | -1.10 | 0.345 | 0.418 | 0.999 | 4428 | tags=23%, list=13%, signal=27% |
| 110 | KEGG\_ANTIGEN\_PROCESSING\_AND\_PRESENTATION |  | 79 | -0.34 | -1.08 | 0.360 | 0.440 | 0.999 | 6396 | tags=19%, list=18%, signal=23% |
| 111 | KEGG\_VEGF\_SIGNALING\_PATHWAY |  | 75 | -0.30 | -1.08 | 0.337 | 0.446 | 1.000 | 4503 | tags=15%, list=13%, signal=17% |
| 112 | KEGG\_CALCIUM\_SIGNALING\_PATHWAY |  | 177 | -0.28 | -1.07 | 0.294 | 0.454 | 1.000 | 5695 | tags=21%, list=16%, signal=25% |
| 113 | KEGG\_ERBB\_SIGNALING\_PATHWAY |  | 86 | -0.24 | -1.06 | 0.373 | 0.461 | 1.000 | 6814 | tags=20%, list=20%, signal=25% |
| 114 | KEGG\_LINOLEIC\_ACID\_METABOLISM |  | 28 | -0.40 | -1.06 | 0.365 | 0.460 | 1.000 | 5345 | tags=25%, list=15%, signal=30% |
| 115 | KEGG\_NUCLEOTIDE\_EXCISION\_REPAIR |  | 43 | -0.25 | -1.05 | 0.395 | 0.469 | 1.000 | 13639 | tags=58%, list=39%, signal=96% |
| 116 | KEGG\_SELENOAMINO\_ACID\_METABOLISM |  | 24 | -0.35 | -1.05 | 0.418 | 0.478 | 1.000 | 8365 | tags=29%, list=24%, signal=38% |
| 117 | KEGG\_N\_GLYCAN\_BIOSYNTHESIS |  | 46 | -0.32 | -1.04 | 0.432 | 0.485 | 1.000 | 12186 | tags=33%, list=35%, signal=50% |
| 118 | KEGG\_PPAR\_SIGNALING\_PATHWAY |  | 69 | -0.33 | -1.03 | 0.397 | 0.488 | 1.000 | 8755 | tags=33%, list=25%, signal=44% |
| 119 | KEGG\_RETINOL\_METABOLISM |  | 55 | -0.32 | -1.03 | 0.378 | 0.484 | 1.000 | 2884 | tags=16%, list=8%, signal=18% |
| 120 | KEGG\_MISMATCH\_REPAIR |  | 23 | -0.30 | -1.03 | 0.394 | 0.481 | 1.000 | 12464 | tags=61%, list=36%, signal=95% |
| 121 | KEGG\_UBIQUITIN\_MEDIATED\_PROTEOLYSIS |  | 130 | -0.18 | -1.02 | 0.400 | 0.496 | 1.000 | 13841 | tags=39%, list=40%, signal=65% |
| 122 | KEGG\_GNRH\_SIGNALING\_PATHWAY |  | 100 | -0.23 | -1.02 | 0.401 | 0.497 | 1.000 | 4503 | tags=12%, list=13%, signal=14% |
| 123 | KEGG\_VALINE\_LEUCINE\_AND\_ISOLEUCINE\_DEGRADATION |  | 43 | -0.28 | -1.00 | 0.477 | 0.526 | 1.000 | 9284 | tags=23%, list=27%, signal=32% |
| 124 | KEGG\_BASE\_EXCISION\_REPAIR |  | 33 | -0.31 | -1.00 | 0.467 | 0.525 | 1.000 | 12458 | tags=45%, list=36%, signal=71% |
| 125 | KEGG\_ARGININE\_AND\_PROLINE\_METABOLISM |  | 52 | -0.28 | -0.99 | 0.476 | 0.524 | 1.000 | 936 | tags=8%, list=3%, signal=8% |
| 126 | KEGG\_MTOR\_SIGNALING\_PATHWAY |  | 51 | -0.28 | -0.99 | 0.433 | 0.530 | 1.000 | 6922 | tags=18%, list=20%, signal=22% |
| 127 | KEGG\_ALPHA\_LINOLENIC\_ACID\_METABOLISM |  | 18 | -0.42 | -0.98 | 0.445 | 0.534 | 1.000 | 757 | tags=11%, list=2%, signal=11% |
| 128 | KEGG\_AUTOIMMUNE\_THYROID\_DISEASE |  | 48 | -0.36 | -0.97 | 0.500 | 0.557 | 1.000 | 6813 | tags=33%, list=20%, signal=41% |
| 129 | KEGG\_HEMATOPOIETIC\_CELL\_LINEAGE |  | 87 | -0.32 | -0.96 | 0.527 | 0.561 | 1.000 | 7312 | tags=34%, list=21%, signal=44% |
| 130 | KEGG\_RENIN\_ANGIOTENSIN\_SYSTEM |  | 17 | -0.41 | -0.95 | 0.530 | 0.570 | 1.000 | 4936 | tags=24%, list=14%, signal=27% |
| 131 | KEGG\_AMINOACYL\_TRNA\_BIOSYNTHESIS |  | 22 | -0.32 | -0.95 | 0.490 | 0.572 | 1.000 | 15880 | tags=68%, list=46%, signal=126% |
| 132 | KEGG\_GLIOMA |  | 65 | -0.23 | -0.94 | 0.555 | 0.579 | 1.000 | 3601 | tags=11%, list=10%, signal=12% |
| 133 | KEGG\_LONG\_TERM\_POTENTIATION |  | 70 | -0.23 | -0.93 | 0.569 | 0.596 | 1.000 | 4098 | tags=13%, list=12%, signal=15% |
| 134 | KEGG\_GLYCEROLIPID\_METABOLISM |  | 43 | -0.26 | -0.90 | 0.623 | 0.652 | 1.000 | 371 | tags=7%, list=1%, signal=7% |
| 135 | KEGG\_ECM\_RECEPTOR\_INTERACTION |  | 84 | -0.27 | -0.88 | 0.587 | 0.686 | 1.000 | 10894 | tags=42%, list=31%, signal=61% |
| 136 | KEGG\_STARCH\_AND\_SUCROSE\_METABOLISM |  | 39 | -0.29 | -0.87 | 0.631 | 0.695 | 1.000 | 3174 | tags=15%, list=9%, signal=17% |
| 137 | KEGG\_OXIDATIVE\_PHOSPHORYLATION |  | 98 | -0.20 | -0.87 | 0.578 | 0.693 | 1.000 | 15058 | tags=35%, list=43%, signal=61% |
| 138 | KEGG\_CITRATE\_CYCLE\_TCA\_CYCLE |  | 30 | -0.21 | -0.86 | 0.623 | 0.704 | 1.000 | 14921 | tags=53%, list=43%, signal=93% |
| 139 | KEGG\_SNARE\_INTERACTIONS\_IN\_VESICULAR\_TRANSPORT |  | 38 | -0.25 | -0.86 | 0.607 | 0.700 | 1.000 | 9630 | tags=24%, list=28%, signal=33% |
| 140 | KEGG\_BETA\_ALANINE\_METABOLISM |  | 22 | -0.32 | -0.85 | 0.665 | 0.706 | 1.000 | 9815 | tags=41%, list=28%, signal=57% |
| 141 | KEGG\_RNA\_POLYMERASE |  | 27 | -0.26 | -0.83 | 0.723 | 0.732 | 1.000 | 14822 | tags=52%, list=43%, signal=90% |
| 142 | KEGG\_OLFACTORY\_TRANSDUCTION |  | 372 | -0.25 | -0.83 | 0.960 | 0.743 | 1.000 | 6209 | tags=26%, list=18%, signal=31% |
| 143 | KEGG\_PROGESTERONE\_MEDIATED\_OOCYTE\_MATURATION |  | 85 | -0.21 | -0.82 | 0.714 | 0.738 | 1.000 | 7953 | tags=20%, list=23%, signal=26% |
| 144 | KEGG\_OTHER\_GLYCAN\_DEGRADATION |  | 15 | -0.28 | -0.82 | 0.642 | 0.736 | 1.000 | 1379 | tags=7%, list=4%, signal=7% |
| 145 | KEGG\_PROPANOATE\_METABOLISM |  | 31 | -0.26 | -0.82 | 0.656 | 0.734 | 1.000 | 10354 | tags=29%, list=30%, signal=41% |
| 146 | KEGG\_NOTCH\_SIGNALING\_PATHWAY |  | 47 | -0.21 | -0.80 | 0.727 | 0.770 | 1.000 | 13023 | tags=45%, list=37%, signal=71% |
| 147 | KEGG\_DRUG\_METABOLISM\_OTHER\_ENZYMES |  | 42 | -0.25 | -0.79 | 0.822 | 0.772 | 1.000 | 9367 | tags=31%, list=27%, signal=42% |
| 148 | KEGG\_PENTOSE\_PHOSPHATE\_PATHWAY |  | 27 | -0.20 | -0.79 | 0.804 | 0.768 | 1.000 | 6000 | tags=15%, list=17%, signal=18% |
| 149 | KEGG\_STEROID\_BIOSYNTHESIS |  | 17 | -0.24 | -0.77 | 0.730 | 0.792 | 1.000 | 3404 | tags=12%, list=10%, signal=13% |
| 150 | KEGG\_BIOSYNTHESIS\_OF\_UNSATURATED\_FATTY\_ACIDS |  | 19 | -0.22 | -0.76 | 0.801 | 0.802 | 1.000 | 7609 | tags=21%, list=22%, signal=27% |
| 151 | KEGG\_REGULATION\_OF\_AUTOPHAGY |  | 30 | -0.25 | -0.76 | 0.782 | 0.805 | 1.000 | 6754 | tags=23%, list=19%, signal=29% |
| 152 | KEGG\_FATTY\_ACID\_METABOLISM |  | 42 | -0.21 | -0.71 | 0.824 | 0.860 | 1.000 | 532 | tags=5%, list=2%, signal=5% |
| 153 | KEGG\_ABC\_TRANSPORTERS |  | 44 | -0.23 | -0.71 | 0.892 | 0.863 | 1.000 | 5067 | tags=18%, list=15%, signal=21% |
| 154 | KEGG\_CYSTEINE\_AND\_METHIONINE\_METABOLISM |  | 34 | -0.15 | -0.66 | 0.912 | 0.911 | 1.000 | 10763 | tags=35%, list=31%, signal=51% |
| 155 | KEGG\_GLYCOSAMINOGLYCAN\_BIOSYNTHESIS\_HEPARAN\_SULFATE |  | 26 | -0.20 | -0.65 | 0.950 | 0.916 | 1.000 | 10813 | tags=38%, list=31%, signal=56% |
| 156 | KEGG\_MATURITY\_ONSET\_DIABETES\_OF\_THE\_YOUNG |  | 25 | -0.25 | -0.64 | 0.971 | 0.916 | 1.000 | 10034 | tags=40%, list=29%, signal=56% |
Table: Gene sets enriched in phenotype **l (8 samples) **[plain text format]****
